# Prevalence and risk factors of feeding difficulties in children with delayed reconstruction of esophageal atresia: a Swedish nationwide study

**DOI:** 10.1007/s00383-025-06052-4

**Published:** 2025-06-11

**Authors:** Sofie Örnö Ax, Elin Öst, Helene Engstrand Lilja, Erik Omling, Vladimir Gatzinsky, Jan F. Svensson, Ann-Marie Kassa, Linus Jönsson, AnnaMaria Tollne, Pernilla Stenström, Kate Abrahamsson, Michaela Dellenmark-Blom

**Affiliations:** 1 Institute of Clinical Sciences, Department of Pediatrics, Queen Silvia Children’s Hospital, Rondvägen 10, 41640 Gothenburg, Sweden; 2 Department of Pediatric Surgery, Queen Silvia Children’s Hospital, Sahlgrenska University Hospital, Gothenburg, Sweden; 3 Department of Pediatric Surgery, Karolinska University Hospital, Stockholm, Sweden; 4 Department of Women’s and Children’s Health, Karolinska Institutet, Stockholm, Sweden; 5 Department of Women’s and Children’s Health, Uppsala University, Uppsala, Sweden; 6 Department of Pediatric Surgery, University Children’s Hospital, Uppsala, Sweden; 7 Department of Pediatric Surgery, Lund University, Skane University Hospital, Lund, Sweden

**Keywords:** Esophageal atresia, Morbidity, Feeding, Surgical outcomes

## Abstract

**Purpose:**

Children with delayed reconstruction of esophageal atresia (DREA) have a high risk of morbidity. The aim of this study was to determine the prevalence of feeding difficulties in children with DREA, differences to children with primary anastomosis (PA) and identify risk factors for feeding difficulties in children with DREA.

**Methods:**

Parents of 30 children with DREA, born 2001–2018, were recruited nationwide in Sweden and answered an author-developed screening survey about feeding difficulties. Outcomes were statistically compared to 105 children with PA, who were hypothesized to be more mildly affected Relative risk factors for feeding difficulties were investigated using negative binominal regression. Clinical data was retrieved from medical records. Level of significance *p* < 0.05.

**Results:**

A majority of children with DREA (83%) had feeding difficulties, most commonly “longer mealtimes than peers” (73%) and “cough/choking during meals” (72%). Food infusion pump use (*p* = 0.043), need to avoid specific foods (*p* = 0.049) and need to eat with extra support by an adult (*p* < 0.0001) were more frequent than in PA. The relative risk of feeding difficulties increased with younger child age (*p* = 0.016), number of associated anomalies (*p* = 0.04) number of respiratory (*p* = 0.002) and digestive symptoms (*p* = 0.005).

**Conclusion:**

Feeding difficulties in children with DREA are prevalent, underlining the need for multidisciplinary and targeted care early in life.

**Supplementary Information:**

The online version contains supplementary material available at 10.1007/s00383-025-06052-4.

## Background

Esophageal atresia (EA), is a rare congenital discontinuity of the esophagus affecting 2.43/10,000 newborns [1]. In high income countries survival nowadays exceeds 90% [2]. A primary anastomosis (PA) of the esophageal ends is reported to be unfeasible in 10% of newborns with EA [3]. These children later undergo a delayed reconstruction of EA (DREA). The most common reason for DREA is a long distance between the esophageal ends, referred to as long-gap EA (LGEA) [3]. Other reasons cited are frailty of the newborn because of low birth weight and/or prematurity [4]. There is no gold standard operative strategy for DREA. A delayed primary anastomosis (DPA) can be performed in some infants with LGEA after initial insertion of a gastrostomy tube for enteral feeding followed by waiting for some months for growth of the child and the esophageal segments [5]. Other strategies include the use of elongation traction technique [6, 7] or esophageal replacement(ER). ER with either stomach, jejunum, colon or a gastric tube is used when the gap is too wide to perform a DPA, or as revisional surgery[8].

It is well known that the rate of esophageal- and respiratory morbidity is elevated among children with EA [9, 10]. In children with DREA, previous studies indicate an even stronger association with long-term gastrointestinal sequelae such as anastomotic strictures, gastro-esophageal reflux disease and feeding difficulties [3, 11–15]. In a recent meta-analysis, the pooled prevalence of studied mealtime adaptations for children with EA encompassed a need to eat slowly (37%), to drink fluid to facilitate eating (49%) and to modify textures(28%). Moreover, eating impairments such as difficulty swallowing solids and odynophagia were reported in 45% and 30% of cases, respectively [16]. Feeding difficulties among children with EA are shown to be associated with chronic respiratory symptoms [17] and may play a role in growth outcomes [18]. As eating forms a crucial component of disease-specific health-related quality of life (HRQOL) in children with EA [19–21], there is a distinct association of feeding related morbidity and of caregivers’ anxiety, fear, isolation and sadness [22–25]. Therefore, more knowledge of feeding difficulties in children with DREA is warranted.

The first aim of this study was to screen for the prevalence of feeding difficulties in a nationwide recruited cohort of pediatric patients with DREA in Sweden. Second, this study aimed to determine if feeding difficulties among children with DREA differ from children with PA, and the third aim was to identify risk factors associated with feeding difficulties in children with DREA.

## Material and methods

This manuscript is part of a nationwide research project in Sweden that explores postoperative morbidity, HRQOL and schooling experiences in children treated with DREA [26–28]. Ethical approval was given by the Swedish Ethical Committee in 2019 (2019-04,930) and 2020(2020-04,310). Written informed consent for study participation was obtained from parents for all child ages and from children at 15 years of age and older.

### Assessment of digestive, respiratory and feeding difficulties

A standardized survey developed by the authors collected information on the child’s symptoms in the last 4 weeks and included digestive symptoms and respiratory symptoms. Nine questions regarding the presence of feeding difficulties in children born with EA over 4 weeks, as seen from the parents’ perspectives, were developed from a review of the literature in EA and discussed in a multidisciplinary team. The questions were shown to be feasible as applied in earlier studies published by our group [29, 30] and enabled comparison of outcomes of children with PA. In addition, in the nationwide study of children with DREA, six parent-reported questions about swallowing difficulties and food aversion were added (Supplemental material 2 and 3).

#### Children with DREA

DREA was defined as when a PA was unattainable during the first surgery, either due to LGEA or frailty because of the infant’s low weight, prematurity and/or other co-morbidities. This meant that all these children’s feeding started with enteral feeding and a delayed establishment of oral feeding. Nationwide recruitment of children with DREA was undertaken through collaboration with all four pediatric surgical centers in Sweden. As detailed in our earlier study [26], children with DREA were considered eligible for recruitment if the child was between 2 and 18 years of age at the time of the study and had a family that was proficient in written and spoken Swedish. Altogether, 45 children born between 2001 and 2018 who underwent DREA were identified through hospital records at the Karolinska University Hospital, Stockholm (*n* = 15), the Uppsala University Hospital, Uppsala (*n* = 14), Sahlgrenska University Hospital, Gothenburg (*n* = 13) and Skåne University Hospital, Lund (*n* = 3). Out of the 45 families, one child had died, and three children were lost to follow-up. Two more families were excluded from the study, five declined study participation and four families did not return written informed consent. At the end, 30 (67%) families could be included in the study as they gave written informed consent, completed and returned the requested questionnaires.

The surgical methods for DREA employed in the study sample were divided into the following; DPA (*n* = 14), [31] where esophageal continuity is achieved in a single surgical session much like the one for PA; ER included gastric pull-up (*n* = 3), [32, 33]; partial gastric pull-up (*n* = 3) [34]; gastric tube esophagoplasty (*n* = 8) [35]; and colon interposition (*n* = 2) [36]. In 4 cases, the reconstruction was delayed due to prematurity/low birth weight only, with a delay of median 137 days (range 34–1221 days). For the 26 patients whose gap length was the reason for DREA, the median time to surgery was 103 days (range 36–207 days).

#### Reference population for comparison

One hundred five children with EA (2–18 years, Gross type C) that underwent PA were included as a reference for comparison from Sahlgrenska University Hospital, Gothenburg. They had participated in earlier studies of generic HRQOL [37], field tests of a condition-specific HRQOL-instrument [38, 39] and studies about feeding difficulties [29, 30].

#### Clinical characteristics of children with DREA and PA

The clinical characteristics of children with DREA and the reference population has been described earlier for children aged 2–7 and 8–17 years old [26]. The median age of the children at the time of the study did not differ significantly between the groups (DREA, *n* = 30, median 11 years, range 3–17 vs PA, *n* = 105, median 8 years, range 2–17, *p* = 0.12). Table 1 presents the sample of children treated with DREA and PA in the total group of children aged 2–18 years. As shown, children with DREA were found to be more commonly born prematurely, with birth weight < 2500 g and with anastomotic leakage or sepsis following reconstructive surgery than for children with PA. The prevalence of digestive- and airway symptoms during the last 4 weeks among children with DREA was reported in a previous study [26].

**Table 1:** Descriptive data of children treated with delayed reconstruction of esophageal atresia (*n* = 30) and the reference population of children treated with primary anastomosis (*n* = 105) [26]

	*n*_tot_	Children with delayed reconstruction of esophageal atresia; *n*(%)	*n*_tot_	Children with primary anastomosis of esophageal atresia; *n*(%)	*p* value following significance test using Fisher’s exact test
Neonatal/birth characteristics					
Male sex	30	15 (50)	105	63 (60)	0.40
Gross subtype	30	A = 12 (40) B = 8 (27) C = 10 (33)	105	C = 105 (100)	
Birth weight in grams, median (range)	30	2133 (525–3225)	101	2688 (1070–4260)	
Low birth weight, < 2500 g	30	20 (67)	101	39 (39)	0.011
Gestational age in weeks, median (range)	30	34 (24–40)	103	38 (28–43)	
Prematurely born	30	18 (60)	103	36 (35)	0.020
Associated anomalies	30	20 (66.7)	105	62 (59)	0.53
Cardio-vascular	30	10 (33.3)	105	30 (28.6)	
Anorectal	30	7 (23.3)	105	10 (9.5)	
Other gastro-intestinal	30	3 (10)	104	9 (8.7)	
Uro-genital	30	11 (36.7)	105	12 (11.4)	
Limb	30	2 (6.7)	105	8 (7.6)	
Vertebrae	30	5 (16.7)	105	23 (21.9)	
Choanal atresia	30	1 (3.3)	105	1 (1.0)	
Eye	30	2 (6.7)	105	6 (5.7)	
Ear	30	1 (3.3)	105	3 (2.9)	
Central nervous system	30	2 (6.7)	105	10 (9.5)	
Respiratory	30	2 (6.7)	105	4 (3.8)	
Other	30	2 (6.7)	105	1 (1.0)	
VACTERL*	30	6 (20.0)	105	17 (16.1)	
Number of associated anomalies, median (range)	30	1 (0–6)	104	1 (0–7)	
Verified genetic deviation	30	5 (16.7)	105	10 (9.5)	
Surgical data					
Major revisional surgery	30	5 (17)	105	10 (9.5)	0.32
Anastomosis leakage	30	8 (27)	103	10 (9.7)	0.014
Sepsis verified by blood culture	30		103	14 (13.6)	0.028
Days to reconstruction median (range)	30	184 (34–1221)			
Days to discharge median (range)	30	241 (62–1235)			
Fundoplicatio	30	9 (30)			
PPI-treatment	27	19 (70.4)			

*VACTERL-association, vertebral defects, anal atresia, cardiac defects, esophageal atresia, tracheo-esophageal fistula, renal anomalies, and limb abnormalities

### Data analysis

Statistical data were analyzed using IBM SPSS Statistics for Windows (version 29.0, Armonk, NY, USA: IBM Corp) and SAS software version 9.4 (SAS Institute Inc., Cary, NC, USA). Descriptive statistics were used to present the study population. For categorical variables, frequencies and percentages were used, whereas for continuous variables, median and range were analyzed. Where feeding difficulties had been evaluated in both children with DREA and PA, the differences in prevalence were investigated using Fisher’s exact test for binary variables and Pearson Chi-Square if there were more than two categories. Mann–Whitney *U*-tests were used to analyze differences between two groups when the dependent variable was continuous. To synchronize with our earlier investigation we regarded the number of feeding difficulties in a child with DREA as a main outcome of investigation [29, 30]. We used negative binomial regression for number of feeding difficulties to investigate its relative risk in relation to neonatal/birth data (gestational age, birth weight, associated anomalies), surgical complications after reconstructive surgery (anastomotic leakage, major revisional surgery, sepsis verified by blood culture) as well as characteristics of the child at follow-up (age, number of respiratory or digestive symptoms, respectively). From the negative binomial regression, the relative risk as presented with 95% confidence interval (CI). Due to the low sample size in children with DREA, a multivariable regression analysis and adjustment for possible confounders could not be executed. All tests were two-tailed. Significant level was considered as *p* < 0.05.

## Results

### Prevalence of fifteen feeding difficulties in children with DREA

**Fig. 1:**
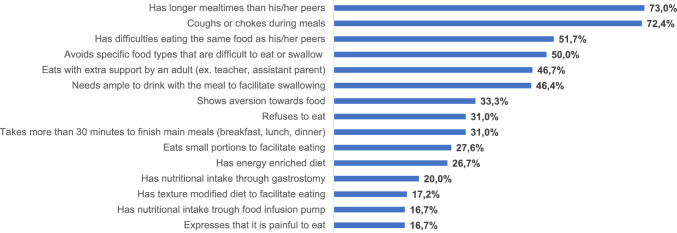
Frequency of feeding difficulties among Swedish children with delayed repair of esophageal atresia (*n* = 30)

Out of the 15 investigated aspects of feeding difficulties listed in Fig. 1, 25 children with DREA were reported to have at least one feeding difficulty during the past month. Most commonly, children with DREA reported longer mealtimes than peers and experiencing coughing or choking during meals. Among the least reported feeding difficulties were the experience of painful mealtimes, texture modified mealtimes and food through infusion pump.

### Comparison of nine feeding difficulties between children treated with DREA and children treated with PA

**Fig. 2:**
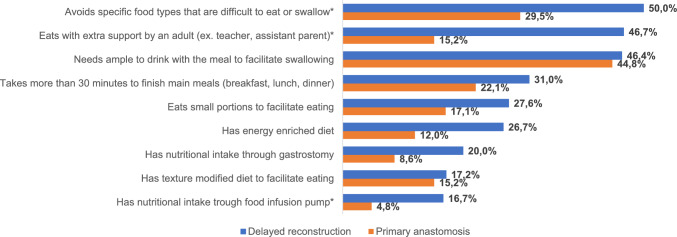
Frequency of nine feeding difficulties in children with delayed (*n* = 30) compared to primary (*n* = 105) reconstruction of esophageal atresia. (*Significant differences in prevalence between children treated with DREA and the reference population of children treated with PA)

In children with DREA 19/30 had at least one of the nine feeding difficulties that were compared to children with PA. Two families of children that were fed with infusion pump at the time for the study did not fully complete the feeding difficulties questionnaire. Missing data is displayed in Supplemental material, Table 1. The median number of feeding difficulties in children with DREA was 2 (range 0–8) and did not differ compared to children with PA (median 1, range 0–9, *p* = 0.25). A significantly higher rate of children with DREA were reported to avoid specific food types that were difficult to eat or swallow compared to children with PA (*p* = 0.049), as well as to have food trough an infusion pump (*p* = 0.043) and to need assistance by an adult at mealtimes (*p* < 0.001). Figure 2. Significant results of the comparison of the surgical subgroups of DREA and PA are displayed in Table 2, wheatears complete results are presented in Supplemental material, Table 1.

**Table 2:** Significant results when comparing the frequency of nine feeding difficulties between the surgical treatment groups of delayed primary anastomosis (*n* = 14), esophageal replacement (*n* = 16) and primary anastomosis (*n* = 105) among children with esophageal atresia*

Frequency in treatment groups *n* (%)		*p* value
“Eats with extra support by an adult (eg., parent, teacher, assistant)”		
Primary anastomosis	Delayed primary anastomosis	
16 (15.2)	7 (50.0)	0.006
Primary anastomosis	Esophageal replacement	
16 (15.2)	7 (43.8)	0.013
“Eats small portions to facilitate eating”		
Primary anastomosis	Delayed primary anastomosis	
18 (17.1)	4 (30.8)	0.031
“Has nutritional intake through gastrostomy”		
Primary anastomosis	Esophageal replacement	
9 (8.6)	5 (31.3)	0.021
“Has nutritional intake through food infusion pump”		
Primary anastomosis	Esophageal replacement	
5 (4.8)	4 (25.0)	0.018

*Complete results in supplemental material; Table 1

### Relative risk for nine feeding difficulties in children with delayed reconstruction of EA

Table 3 presents the findings after negative binomial regression of feeding difficulties in children with DREA. A higher number of associated anomalies was associated to increased relative risk for feeding difficulties (*p* = 0.040), as was lower child age (*p *= 0.016). Children with DREA (n_tot_ = 28) had a median of 2 (0–5) respiratory symptoms and 0 (range 0–3) digestive symptoms. A higher number of respiratory symptoms (*p* = 0.002) and digestive symptoms (*p* = 0.005) significantly increased the relative risk of having feeding difficulties.

**Table 3:** The relationship of neonatal/birth characteristics, complications to reconstructive surgery and respiratory/digestive symptoms at follow-up to the number of feeding difficulties* at follow-up in children with delayed reconstruction of esophageal atresia when using negative binomial regression

	Relative risk (95% CI)	*p* value
Neonatal/birth characteristics		
Birth weight		
Birth weight < 2500 g	1.73 (0.64–4.70)	0.28
Birth weight per 500 g increase	0.90 (0.65–1.23)	0.50
Gestational age		
Prematurely born(< 37 gestational weeks at birth)	0.91 (0.34–2.43)	0.86
Gestational week, per one week increase	0.98 (0.87–1.10)	0.71
Associated anomalies		
Presence of associated anomaly	1.60 (0.59–4.37)	0.35
Number of associated anomalies, per each additional anomaly^1^	1.28 (1.01–1.63)	0.040
Reconstructive surgery for EA		
Major revisional surgery after reconstruction of EA	0.59 (0.16–2.17)	0.43
Anastomotic leakage, conservative treatment or revisional surgery	0.97 (0.35–2.70)	0.95
Sepsis verified by blood culture	1.17 (0.42–3.24)	0.76
Follow-up		
Child age, per one year increase	0.87 (0.78–0.97)	0.016
Respiratory symptoms, per each additional symptom^2^	1.42 (1.14–1.79)	0.002
Digestive symptoms, per each additional symptom^3^	1.80 (1.20–2.72)	0.005

*Nine feeding difficulties, as stated in Fig. 2

^1^Included are cardio-vascular, anorectal, other gastro-intestinal, uro-genital, skeletal-vertebrae anomalies and those of the central nervous system, ear, eye, respiratory or others

^2^Cough, wheezing, respiratory infection, chest tightness, dyspnea the past 4 weeks

^3^Heartburn, vomiting, swallowing difficulties the past 4 weeks

## Discussion

To our knowledge this is the first nationwide study of feeding difficulties in children with DREA. We found a high prevalence (83%) of children with feeding difficulties among children with DREA in Sweden. This was despite the fact that these children had taken part in a standardized follow-up care program to monitor health and treatments. Compared to PA, children with DREA were reported to need assistance at mealtimes significantly more often, to avoid food that is difficult to eat and to use a food infusion pump. The prevalence of feeding difficulties increased with younger child age, the number of associated anomalies and with aerodigestive symptoms.

Already in the early nineties Puntis et al. set the tone for how feeding difficulties can be investigated with questionnaires in children with EA and showed that “long mealtimes” was a major issue [40]*.* In our study, “long mealtimes” was considered by two different questions. “Main mealtimes taking longer than 30 min” had a prevalence in accordance with current literature [16], whereas having “longer mealtimes than peers” had a surprisingly high prevalence(almost three-quarters) in children with DREA. A possible explanation is that it is easier for parents to recognize that their child finishes late at the table, as opposed to quantifying time duration for a mealtime. As having a slower pace when eating has been associated with worse social- and school functioning, negative emotional symptoms [30] and condition-specific HRQOL [38], it warrants special attention in clinical practice. The wide range of prevalence for the different feeding difficulties in EA populations suggests a lack of cohesive terminology and common understanding of what condition-specific eating related morbidity looks like for this patient group [16]. This complicates matters when studying subgroups in the population, such as DREA. Recently, validated feeding scales like the Montreal Children’s Hospital Feeding Scale have been used [17, 41] where children with severe EA were found to have more feeding problems. Yet, generic instruments can miss items which are crucial components of eating related condition-specific HRQOL [19–21] and morbidity [40].

Half of the group of children with DREA reported “difficulties eating the same food as peers” but very few of them needed a modified food texture. A larger proportion of children with DREA than with PA avoided food that was difficult to eat or swallow, which earlier has been defined as a disengagement coping strategy [42] and as a symptom of esophageal dysmotility, stenosis and/or strictures [43]. In agreement with these results, children with DREA are at risk of esophageal stricture [12, 13] and the total number of esophageal dilatations at follow-up for children with DREA was significantly higher than in children with PA in our study population [26]. Esophageal stricture is also a risk factor for “coughing and choking during mealtimes” [43]. Accordingly, coughing and choking during meals was one of the most frequently reported feeding difficulties in this study, and remarkably almost double the estimated pooled prevalence of patients with EA in general [16]. Choking, or the risk of choking, is for parents the most anxiety provoking event when feeding a child with DREA [22, 23]. Yet, the need to increase fluid intake to facilitate swallowing was at the same levels as the PA-group and EA-populations generally [16, 29]. This was unexpected since it is a strategy recommended by caregivers and international support-organizations for EA-families to avoid choking [44, 45].

The greater need for extra adult support at mealtimes in the DREA group compared to PA may suggest a supervision requirement during mealtimes due to safety concerns or the need for gastrostomy-feeding. This was observed in another study, where the majority of children with LGEA needed support with nutritional intake in school [27]. The higher frequency of tube-feeding in the DREA compared to the PA-group could hypothetically explain why more differences between the groups were not found. This was since two families of solely tube-fed children could not answer most feeding difficulty questions.

There were no significant differences when comparing frequencies of feeding difficulties in surgical treatment subgroups within DREA (ER and DPA). This is in agreement with previous results when applying the eating-domain of the EAQOL instrument among the older children in the same cohort [26]. Tube feeding and to have a gastrostomy at follow-up were significantly more frequent in children with ER vs PA, which is important to know as these nutritional needs are associated with worse generic HRQOL [30]. The fact that several feeding difficulties were more common in the DREA treatment groups compared to PA strengthens the hypothesis that with a larger cohort and a validated instrument one would be able to evaluate feeding outcomes following different surgical approaches.

Swallowing difficulties, heartburn and vomiting as well as respiratory symptoms were associated to increased relative risk of concurrent feeding difficulties, which is not surprising. The relative risk of having feeding difficulties also decreased with higher child age. This all agrees with previous research where respiratory morbidity is found to be associated with feeding difficulties, esophageal dysfunction and dysphagia for children with EA [9, 17] and that feeding difficulties are more common in young children with EA [29, 40].

Associated anomalies have been regarded as a possible marker of disease severity of EA [26], and in our study more associated anomalies associated with an increased relative risk of feeding difficulties for children with DREA. Yet, the mere presence of associated anomalies was not related to feeding difficulties for children with neither DREA nor EA [29]. Our results agree with previous research, revealing links between VACTERL association and nutritional intake difficulties in children with EA [29]. Interestingly, children with VACTREL displays feeding problems regardless of the presence of EA [46].

As events that increase the relative risk of feeding difficulties are aligned with previous knowledge, we hypothesize that better knowledge of feeding difficulties in this population can be an accurate tool to determine disease burden. Our results underline the importance of multidisciplinary follow-up, including a dietician, speech and language therapist and a clinical nurse specialist together with a pediatric surgeon, a gastroenterologist and a pulmonologist.

### Study limitations and strengths

The concept DREA encompasses a group where surgical reconstruction was delayed meanwhile the child was fed enterally [26]. Due to retrospective nature of the medical record review, time to oral feeds and how active the oral stimulation was while the child was waiting for reconstruction of the esophagus, could not be identified.

A third of the children with DREA in this study had EA Gross type C, and were compared to a reference population with Type C that were treated with PA. The prevalence of the feeding difficulties that were investigated in this pilot study has previously been found not to be significantly associated to Gross type [29]. It is debated whether the term LGEA should include Gross type A and B regardless of gap length [3] and despite it being a widely used term to describe children with severe EA it has many different definitions [4]. Although patients with DREA represent a heterogenous group, it is a practical approach to indirectly measure disease severity by the initial treatment approach, that enabled us to enlarge the study sample size. A sub-division of the DREA group further than DPA and ER was unfeasible due to small patient groups, even if there is a vast difference of a gastric pull-up and a colonic interposition. Also, our sample size made it unfeasible to take cofounders or child age into consideration in the statistical analysis.

Yet, with a high inclusion rate and a comparison to children that underwent PA, [26] our national cohort study is to our knowledge the largest and first of its kind [16]. A drawback is the lack of a validated questionnaire about feeding difficulties in the Swedish language. Some of the feeding difficulties that had very high prevalence among children with DREA were unfortunately not included in the comparative analysis of children with PA.

### Implications for practice and future research

Recognizing its complexity, enhanced interdisciplinary collaboration—including input from speech and language therapists, dietitians, and psychologists—may support a more comprehensive understanding of the challenges experienced by these patients and their families. Targeted interventions should be considered to start early in life, particularly for children with associated anomalies and persistent aerodigestive morbidity. As an example, pre-operative sham-feed stimulates early oral feeding and may have a positive long-term effect on feeding difficulties [47]. In future research, a patient guided qualitative approach is paramount to capture patients’ and their family’s views on what they find troublesome. To improve detection and management of feeding difficulties, a more structured and diagnose sensitive approach is needed for routine follow-up and long-term care. Incorporating validated, condition-specific questionnaires into clinical practice may facilitate more accurate symptom reporting and could also be used to evaluate surgical methods and other important medical treatment options. This pilot study underlines the need for a validated questionnaire to assess symptom severity for children with EA.

## Supplementary Information

Below is the link to the electronic supplementary material.

Supplementary file1 (DOCX 21 KB)

## Data Availability

No datasets were generated or analysed during the current study.
