# Supplementary material for: Prevalence and risk factors of feeding difficulties in children with delayed reconstruction of esophageal atresia: a Swedish nationwide study
Source: Pediatr Surg Int. 2025 Jun 11;41(1):164. doi: 10.1007/s00383-025-06052-4 (PMC12159094; doi:10.1007/s00383-025-06052-4)
Supplement: Supplementary file 1 — Supplementary file1 (DOCX 21 KB) [file 383_2025_6052_MOESM1_ESM.docx]

*Supplemental material Table 1.*Comparison of eight feeding difficulties between EA treatment-groups among children 2-18 years

|  | **Frequency of feeding difficulties in treatment groups** | | | | | | | |  | **Comparison of treatment groups** | | | |
| --- | --- | --- | --- | --- | --- | --- | --- | --- | --- | --- | --- | --- | --- |
|  | Primary anastomosis | | Delayed reconstruction | | Delayed primary anastomosis | | Esophageal replacement | |  | Primary anastomosis *compared to*  Delayed reconstruction | Primary anastomosis *compared to* Delayed primary anastomosis | Primary anastomosis *compared to* Esophageal replacement | Delayed primary anastomosis *compared to* Esophageal replacement |
| **FEEDING DIFFICLULTY** | n-tot | n(%) | n-tot | n(%) | n-tot | n(%) | n-tot | n(%) |  | p-value, | p-value | p-value | p-value |
| Avoids specific food types that are difficult to eat or swallow | 105 | 31(29.5) | 30 | 15(50.0) | 14 | 8(57.1) | 16 | 7(43.8) |  | **0.049** | 0.07 | 0.26 | 0.72 |
| Eats small portions to facilitate eating | 105 | 18(17.1) | 29 | 8(27.6) | 13 | 4(30.8) | 16 | 4(25.0) |  | 0.29 | **0.031** | 0.49 | 1.00 |
| Has energy enriched diet | 105 | 13(12.4) | 30 | 8(26.7) | 14 | 4(28.6) | 16 | 4(25.0) |  | 0.08 | 0.12 | 0.24 | 1.00 |
| Has texture modified diet to facilitate eating | 105 | 16(15.2) | 29 | 5(17.2) | 14 | 4(28.6) | 15 | 1(6.7) |  | 0.78 | 0.25 | 0.63 | 0.17 |
| Takes more than 30 minutes to finish main meals  (breakfast, lunch, dinner) | 104 | 23(22.1) | 29 | 9(31.0) | 14 | 5(35.7) | 15 | 4(26.7) |  | 0.33 | 0.32 | 0.74 | 0.70 |
| Needs ample to drink with the meal to facilitate swallowing | 105 | 47(44.8) | 28 | 13(46.4) | 13 | 7(53.8) | 15 | 6(40.0) |  | 1.00 | 0.57 | 0.79 | 0.71 |
| Has nutritional intake trough gastrostomy | 105 | 9(8.6) | 30 | 6(20.0) | 14 | 1(7.1) | 16 | 5(31.3) |  | 0.10 | 1.00 | **0.021** | 0.18 |
| Eats with extra support by an adult (ex. teacher, assistant parent) | 105 | 16(15.2) | 30 | 14(46.7) | 14 | 7(50.0) | 16 | 7(43.8) |  | **<0.001** | **0.006** | **0.013** | 1.00 |
| Has nutritional intake through food infusion pump | 105 | 5(4.8) | 30 | 5(16.7) | 14 | 1(7.1) | 16 | 4(25.0) |  | **0.043** | 0.54 | **0.018** | 0.34 |

*Supplemental material Table 2.*

Questions on feeding difficulties that were compared in between children with DREA (n=30) and a reference population of children with EA (gross type C) that underwent primary anastomosis (n=105).

**During the last four weeks, did your child:**

| Avoid certain food types that are difficult to eat or swallow? | Yes/No |
| --- | --- |
| Need ample to drink during meals to facilitate swallowing? | Yes/No |
| Have nutrition/food through a gastrostomy? | Yes/No |
| Have nutritional intake through a food infusion pump?  Need energy enriched food? | Yes/No |
| Need a texture modified diet to facilitate eating? | Yes/No |
| Need support by an adult (ex. teacher, assistant parent) during main meals? | Yes/No |
| Eat small portions to make the mealtimes easier? | Yes/No |
| Take a long time (>30 minutes) to finish main meals (breakfast, lunch, dinner)? | Yes/No |

*Supplemental material Table 3:* Additional questions on feeding difficulties added in the nation-wide study on children treated with DREA (n=30).

**During the last four weeks, did your child:***

| 1. Have longer mealtimes than his/her peers? | Yes/No |
| --- | --- |
| 1. Have difficulties eating the same food as his/her peers? | Yes/No |
| 1. Cough or choke during meals? | Yes/No |
| 1. Refuse to eat? | Yes/No |
| 1. Show aversion towards food? | Yes/No |
| 1. Show signs of that it is painful to eat? | Yes/No |

*Question 1-4 were asked on a scale, which comprised “never, sometimes, 1-2 meals/week, about once a day, more than half of meals, most meals, all meals” and dichotomised to yes/no.
